# Caregiver Burden and an Artifact-Associated Glycemic Instability Cycle in Pediatric Hybrid Closed-Loop Therapy: A Case Report

**DOI:** 10.7759/cureus.106803

**Published:** 2026-04-10

**Authors:** Nicolai Altnickel

**Affiliations:** 1 Clinical Operations, Conclusio - Soziale Dienste GmbH, Iserlohn, DEU; 2 Care Management, Waldstadt-HealthCare GmbH, Iserlohn, DEU

**Keywords:** alarm fatigue, automated insulin delivery, camaps fx, caregiver burden, hybrid closed loop, pediatric type 1 diabetes, school-based diabetes care, sensor artifacts

## Abstract

Hybrid closed-loop (HCL) systems improve glycemic outcomes in pediatric type 1 diabetes but transform rather than eliminate caregiver burden in selected real-world contexts. This case-based analysis describes a selected 14-day real-world observation of a 10-year-old child using an HCL system. Despite improved glycemic stability, active nocturnal interventions were required in 85.7% of observed nights. A recurring pattern, described here as an "artifact-associated glycemic instability cycle," was identified in this single case as a hypothesis-generating observation: physical pressure-induced sensor inaccuracies triggered automated insulin suspension, subsequently leading to rebound hyperglycemia and requiring 18 manual corrections. Frequent and repetitive alarms resulted in sustained cognitive load and features suggestive of alarm fatigue. Contextual environmental factors, such as local cooling, further influenced sensor accuracy and system behavior. These findings emphasize that HCL burden shifts toward a high-vigilance supervisory role. Improving sensor artifact detection and alarm specificity is essential for reducing caregiver strain and enhancing the real-world usability of automated insulin delivery systems in pediatric care.

## Introduction

The management of pediatric type 1 diabetes has been significantly advanced by the introduction of hybrid closed-loop (HCL) systems, which have demonstrated improved glycemic control and increased time-in-range compared to conventional therapy in controlled trials and real-world cohorts [1]. These systems combine real-time continuous glucose monitoring (rtCGM), an insulin pump, and an automated algorithm that adjusts insulin delivery based on sensor glucose values; time-in-range refers to the proportion of time glucose levels remain within the recommended target range. Despite these clinical benefits, real-world use indicates that caregiver involvement remains essential, particularly in pediatric settings where continuous supervision and decision-making are required [2].

A critical aspect of HCL use is the occurrence of sensor artifacts - temporary inaccuracies caused by physical pressure or environmental factors - which can trigger inappropriate automated system responses [3]. When these artifacts occur, the system may suspend insulin delivery based on false-low readings, leading to subsequent rebound hyperglycemia and requiring manual intervention [4].

We identify this recurring pattern as an "artifact-associated glycemic instability cycle," which contributes to sustained cognitive load and phenomena consistent with alarm fatigue [5]. While clinical trials report aggregated outcomes, the practical management of such technical disruptions remains less well characterized [6]. This case report describes a 14-day observation of a 10-year-old child, focusing on the interaction between these sensor-related events and caregiver workload [7].

## Case presentation

The patient is a 10-year-old male born in December 2015, diagnosed with type 1 diabetes in 2022. Following the initial diagnosis, the patient was managed using intensive insulin therapy (ICT) with a basal-bolus regimen consisting of Protaphane and NovoRapid. During the first 15 months of treatment, rtCGM was performed using multiple systems, including FreeStyle Libre 3, Dexcom G6, and Dexcom G7.

In mid-2023, the patient transitioned to HCL therapy to improve glycemic control and address reduced hypoglycemia awareness. The current system consists of a mylife YpsoPump, the CamAPS FX automated insulin delivery algorithm, and a FreeStyle Libre 3 sensor.

A structured 14-day real-world observation was conducted during routine daily life, including school attendance and nocturnal home care. This represents a short observational window and limits generalizability. During this period, overall glycemic stability improved, with high time-in-range observed. However, caregiver involvement remained substantial.

Active nocturnal interventions were required in 12 of 14 nights (85.7%), with cumulative management periods of approximately one to two hours per episode.

A recurring pattern of glycemic instability was observed in association with sensor-related inaccuracies, particularly pressure-induced events (“compression lows”) and, in some instances, environmental factors such as local cooling. These events were associated with temporarily low glucose readings, which prompted automated insulin reduction or suspension. Following resolution of these events, rebound hyperglycemia was frequently observed.

In multiple instances, basal adjustments alone were insufficient to correct these excursions, and manual correction boluses were required. A total of 18 manual correction boluses were administered during the observation period.

In practice, these events required caregivers to reposition the child, reassess glucose trends, and perform extended monitoring after corrective interventions. Post-intervention observation periods typically lasted 90-120 minutes, as corrective insulin administration occurred in the context of prior insulin reduction or suspension. With subsequent normalization of sensor readings, automated insulin delivery resumed, potentially contributing to cumulative insulin exposure. This required extended monitoring due to the risk of delayed hypoglycemia.

Frequent system-generated alerts were observed, particularly during nighttime hours, often occurring in clustered patterns. Caregivers were required to repeatedly assess and differentiate between clinically relevant alarms and those associated with sensor-related inaccuracies. This resulted in sustained cognitive load and progressive desensitization to repeated alerts.

Caregiving demands also affected daily routines and included sleep disruption, resistance to nocturnal interventions, and limitations in regular activities.

For the purpose of this report, “manual system entries” refers to caregiver-initiated system interactions other than manual correction boluses, such as carbohydrate entries and bolus-related inputs. A quantitative summary of caregiver burden and system interaction during the observation period is provided in Table 1.

**Table 1:** Caregiver burden and system interaction (14-day observation period)

Category	Parameter	Data
Nocturnal Burden	Nights with intervention	12/14 (85.7%)
	Active nighttime care duration	1–2 hours per episode
Alarm Load	Frequency	Recurrent clustered alerts (nocturnal)
Glycemic Control	Hypoglycemia (<70 mg/dL)	14 events (lowest: 39 mg/dL)
	Hyperglycemia (>250 mg/dL)	12 events (peak: 419 mg/dL)
System Interaction	Manual system entries	>85
	Manual correction boluses	18
Technical Load	Infusion set changes	5
Psychosocial Impact	Behavioral resistance and sleep disruption	Frequently present

This is a single-case observation of a minor patient conducted in a private home setting. Informed consent for the collection and publication of de-identified data was obtained from the legal guardians. The observation involved routine care and did not include experimental interventions or changes to prescribed medical protocols. Therefore, according to local regulations and the nature of the study, formal Institutional Review Board (IRB) approval was not required.

## Discussion

This case report demonstrates that HCL therapy can improve glycemic stability while not eliminating caregiver burden in pediatric type 1 diabetes. Instead, caregiving appears to shift from direct insulin administration toward continuous supervision, interpretation of system behavior, and management of system-related events.

A key observation in this case was a recurring pattern of glycemic instability associated with sensor-related inaccuracies, particularly pressure-induced artifacts (“compression lows”) and, in some instances, environmental factors such as local cooling. These events were associated with temporarily low glucose readings, followed by automated insulin reduction or suspension. Subsequent rebound hyperglycemia was frequently observed after resolution of these events, possibly reflecting a preceding relative insulin deficit [3]. In multiple instances, additional manual intervention was required to restore glycemic control.

**Figure 1:**
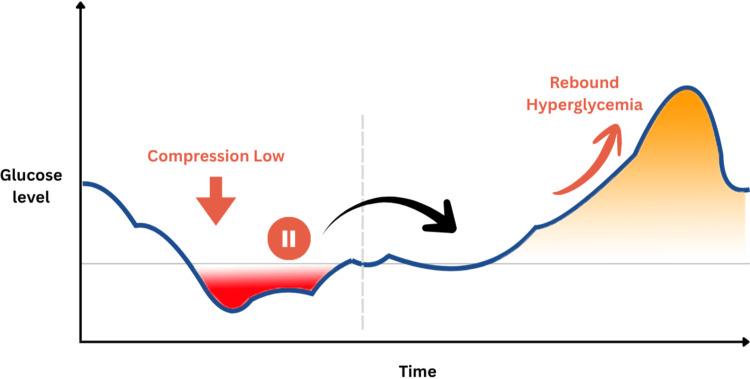
Schematic representation of an artifact-associated insulin suspension pattern in hybrid closed-loop therapy The x-axis represents time, and the y-axis represents glucose level. The blue curve illustrates the sensor-derived glucose trajectory. The red shaded area highlights a non-physiological decrease in glucose values due to a sensor artifact (“compression low”), rather than true hypoglycemia. This falsely low reading triggers automated insulin reduction or suspension by the hybrid closed-loop system. The orange circular symbol with two vertical bars indicates this automated reduction or temporary suspension of insulin delivery in response to the erroneous low sensor values. Following resolution of the sensor artifact, a relative insulin deficit occurs, leading to a progressive rise in glucose levels. The orange shaded area represents the resulting rebound hyperglycemia. Arrows indicate the temporal and causal sequence from sensor artifact to insulin suspension and subsequent hyperglycemia. This schematic illustrates the interaction between sensor-related inaccuracies, algorithm-driven insulin modulation, and subsequent glycemic dynamics in real-world hybrid closed-loop use. Author-created schematic figure produced using Canva (Canva Pty Ltd., Australia) with no AI-generated or AI-modified content.

This observed sequence is illustrated schematically in Figure 1.

Although HCL systems are designed to respond safely to low glucose values, these findings suggest that system behavior in real-world settings may be influenced by contextual factors that are not directly related to true glycemic physiology. The resulting need for repeated manual corrections and extended monitoring periods contributed to sustained caregiver involvement.

A particularly relevant finding was the post-intervention monitoring requirement. Following manual correction of hyperglycemia, caregivers frequently performed extended observation for 90-120 minutes due to the risk of delayed hypoglycemia. This contributed to sustained cognitive load and sleep disruption [5].

Alarm burden emerged as an additional factor. Frequent system-generated alerts, particularly during nighttime hours, required continuous evaluation and prioritization. Repeated exposure to such alerts was associated with observable desensitization consistent with alarm fatigue, a phenomenon described in other medical monitoring contexts [6]. In pediatric care settings, this may have implications for sustained vigilance over time.

In addition to pressure-related artifacts, contextual influences such as local temperature and peripheral perfusion appeared to affect sensor readings in individual situations; however, this should be regarded as a speculative observation requiring further validation. These observations suggest that current rtCGM-based systems may be sensitive to environmental and physiological conditions that are not fully accounted for in algorithmic decision-making.

Taken together, these findings indicate that while HCL systems improve metabolic outcomes, they may shift caregiver responsibilities toward continuous system oversight. This shift may be particularly relevant in pediatric care, where caregivers remain central to decision-making and safety. A directly comparable pre-HCL/ICT dataset using the same structured observational framework was not available; therefore, formal baseline comparison was not possible.

Potential implications include improved recognition of sensor-related inaccuracies, context-aware interpretation of rtCGM data, and optimization of alarm systems to reduce unnecessary alerts [7]. However, further investigation in larger cohorts is required to better understand the frequency and clinical relevance of these observations.

These observations may also have implications for future sensor and algorithm development. One potential approach could be the integration of additional contextual sensing modalities, such as pressure- or temperature-related inputs into rtCGM-based systems, although the practical feasibility, hardware integration, and clinical validation of such approaches remain uncertain. In principle, such information could help identify conditions associated with sensor-related inaccuracies and improve the interpretation of implausible glucose trends. Further studies could evaluate whether correlations between local pressure, temperature, and falsely low glucose readings can be characterized in a clinically useful manner. If feasible, such strategies may reduce unnecessary alerts, improve system robustness, and lessen caregiver burden, including the risk of alarm fatigue.

## Conclusions

HCL therapy improved glycemic stability in this pediatric case but did not eliminate caregiver burden. Instead, caregiving shifted toward continuous supervision, interpretation of system behavior, and management of system-related events.

Sensor-related inaccuracies were associated with repeated episodes of glycemic instability, including insulin reduction or suspension followed by rebound hyperglycemia, which required manual intervention and extended monitoring. These events contributed to sustained caregiver involvement and were accompanied by increased cognitive load and frequent exposure to system-generated alerts consistent with alarm fatigue.

This case suggests that glycemic outcome measures alone may not fully reflect the practical demands of HCL therapy in real-world pediatric settings. Consideration of caregiver workload, system interaction, and alarm burden may provide a more comprehensive assessment of treatment impact.

Further investigation is required to better understand these observations and to explore potential strategies for improving system usability and reducing caregiver burden.
